# Malignant Proliferating Trichilemmal Tumor: A Subtle Presentation in an African American Woman and Review of Immunohistochemical Markers for This Rare Condition

**DOI:** 10.7759/cureus.17289

**Published:** 2021-08-18

**Authors:** Tejas P Joshi, Sharon Marchand, Jaime Tschen

**Affiliations:** 1 Dermatology, Baylor College of Medicine, Houston, USA; 2 Dermatology, Dermatology and Laser Medicine of Southeast Texas, Beaumont, USA; 3 Dermatology, St Joseph Dermatopathology, Houston, USA

**Keywords:** proliferating trichilemmal cyst, proliferating trichilemmal tumor, malignant proliferating trichilemmal cyst, malignant proliferating trichilemmal tumor, cluster of differentiation 34

## Abstract

A malignant proliferating trichilemmal tumor (MPTT) is thought to represent the malignant counterpart of a benign proliferating trichilemmal cyst, a keratin-filled lesion that derives from the outer hair root sheath. The clinical appearance of MPTTs does not always correlate with their histopathologic behavior, emphasizing the need for biopsy and histopathological analysis. Here, we present a 46-year-old African American woman who was evaluated for an ostensibly benign cyst on her scalp that was diagnosed as an MPTT following histopathological examination. She was treated with simple surgical resection that was flush with the cyst margins, followed by Mohs surgery to ensure complete resection. As immunohistochemistry (IHC) has often been used to characterize MPTTs, we also review the various IHC markers reported in the literature.

## Introduction

A proliferating trichilemmal cyst is a keratin-filled lesion that derives from the outer hair root sheath. Usually, proliferating trichilemmal cysts are benign, although rarely, can undergo malignant transformation, at which point they are referred to as malignant proliferating trichilemmal tumors (MPTTs) [1].

Clinically, MPTTs classically present as rapidly growing masses accompanied by necrosis of underlying tissue; the scalp is a common site of MPTTs. Histologically, MPTTs are characterized by abrupt keratinization, anaplastic and pleomorphic cells, an increased number of mitotic figures that may be aberrant, and infiltrative growth [1].

There is no established paradigm of care for MPTTs, though surgical resection with a 1-centimeter margin has been generally recommended. Chemotherapy may be indicated for the management of metastases [2]. Here, we present a 46-year-old African American woman with MPTT that had a seemingly benign clinical presentation. We also review the literature to discuss the immunohistochemistry (IHC) of MPTTs.

## Case presentation

A 46-year-old African American woman presented with a 1.5 x 1.2-centimeter painless cyst on her scalp. The patient reported having had the cyst for many years and mentioned that the cyst would undergo fluctuations in size, enlarging and shrinking intermittently. The epidermis overlying the cyst was unremarkable: no hyperpigmentation, induration, or necrosis was observed. The patient recalled no history of trauma to the lesion.

The patient has no history of any malignancies. Family history is negative for MPTT. She has diabetes mellitus, hypercholesterolemia, and hypertension, managed with metformin, simvastatin, and lisinopril, respectively. She denies any tobacco or alcohol use. The patient had undergone a hysterectomy and a patent foramen ovale closure.

**Figure 1:**
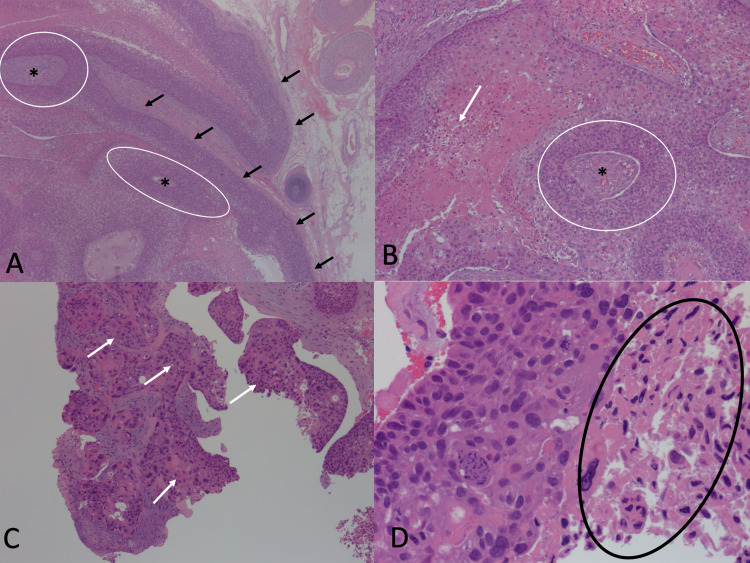
Histopathology of 1.5 x 1.2-centimeter biopsy of the scalp. (A) Circles surround epithelial nests. Asterisks indicate central keratinization. Black arrows point to areas of abrupt keratinization. (B) Arrow points to the area of necrosis. Asterisk indicates central keratinization. A circle surrounds an epithelial nest. (C) Arrows point to regions of frank atypia. (D) A circle surrounds the region with abnormal mitotic figures.

She desired surgical excision of the cyst because she felt it to be a minor nuisance. No picture was taken of the cyst prior to surgical resection as there was no clinical suspicion for an MPTT. A simple surgical excision was performed that was flush with the margins of the cyst. Histopathological examination of the biopsy revealed epithelial nests with abrupt keratinization characteristic of trichilemmal epithelium. Tumor necrosis, anaplastic and pleomorphic cells, and increased mitotic figures were also observed, consistent with a diagnosis of MPTT. Margins were positive for atypia (Figures 1A-1D).

**Figure 2:**
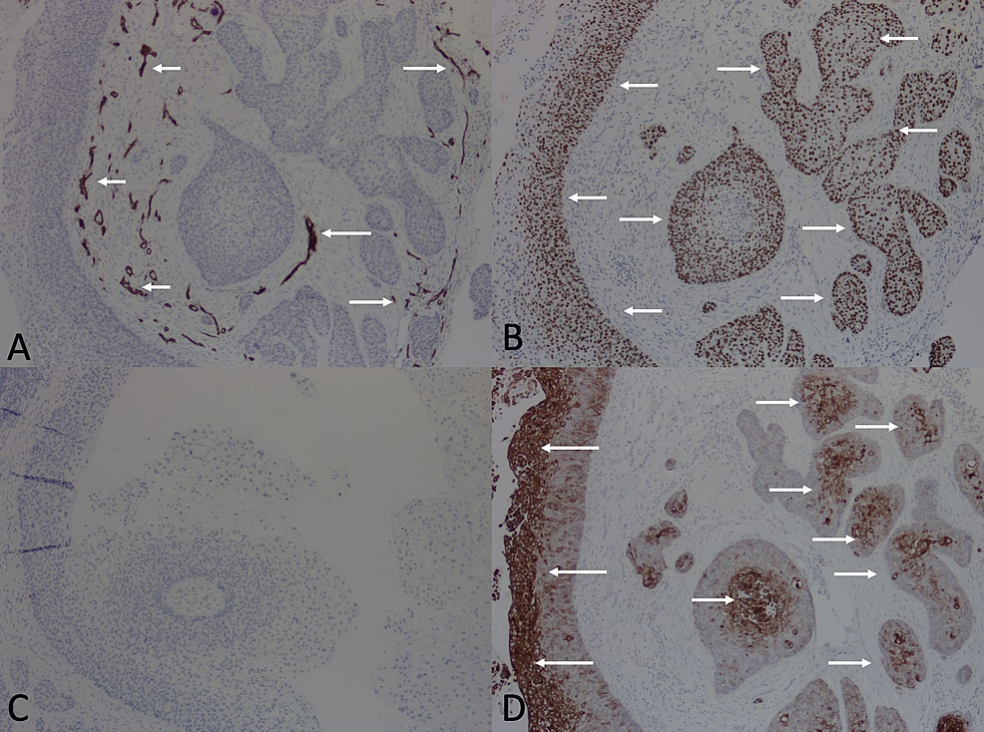
Immunohistochemistry of 1.5 x 1.2-centimeter biopsy of the scalp. (A) Arrows point to areas of CD34 expression, which is limited to blood vessels (positive internal control). The tumor is otherwise negative for CD34 expression. (B) Arrows point to areas expressing p53. (C) No CK 7 expression is detected. (D) Arrows point to areas expressing CK 17. Abbreviations: CD, cluster of differentiation; CK, cytokeratin

IHC studies were also performed. The tumor was negative for a cluster of differentiation (CD) 34 (only blood vessels stained with CD34, serving as positive internal controls). Staining with p53, a proliferation marker, was strongly positive. Additionally, pronounced cytokeratin 17 (CK 17) expression was observed, although CK 7 expression was not detected (Figures 2A-2D).

Following the histopathological diagnosis, the patient was referred to Mohs surgery for resection of a wider margin to remove any malignant tissue that may have been missed in the initial resection. A head and neck computed tomography was performed and was normal. For the next two years, we will follow-up with the patient every three months to inspect her scalp, neck, and lymph nodes; if no lesions appear within the next two years, we will monitor the patient every six months for three years, after which we plan to perform annual check-ups.

## Discussion

The first report of a pilar cyst is credited to Wilson-Jones, who described the lesion in 1966 [3]. In 1983, Saida et al. reported the first case of an MPTT and distinguished MPTTs from benign trichilemmal tumors based on the degree of atypia and mitotic activity [4]. In 2004, Ye et al. analyzed 76 cases of proliferating trichilemmal tumors (PTTs) to propose a classification scheme. The investigators considered well-circumscribed lesions without mitotic figures, necrosis, or neural and vascular invasion to be benign, Group I PTTs. Lesions that infiltrated the deep dermis and subcutis were classified as Group II PTTs and deemed to have the potential for locally aggressive growth. Lesions with pronounced nuclear atypia, increased mitotic figures, and necrosis were assigned to be Group III PTTs and regarded as having metastatic potential [5].

Clinically, MPTTs typically present as rapidly growing, exophytic lesions that are usually greater than three centimeters in size at the time of presentation. MPTTs tend to occur on the scalp and have a predilection for women in their seventh and eighth decades of life [1]. Given the rarity of the condition, it is unclear if MPTT also has a predilection for any particular race. Our case, which describes an MPTT occurring in an African American woman, highlights that MPTT can also occur among African Americans.

Histologically, MPTTs present with anaplastic and pleomorphic cells, heterochromatic nuclei, and increased mitotic forms. Abrupt keratinization (transition from a nucleated epithelial layer to an anucleate keratinized layer without an intervening stratum granulosum) is considered to be the pathologic hallmark of MPTTs. Vascular and neural invasion may also be observed [1].

Interestingly, as was the case in our patient, histopathological behavior sometimes does not correlate with clinical presentation. That is, an ostensibly benign PTT may display marked atypia under pathological examination, and vice versa. Indeed, the lack of a clinicopathological correlation has led some to suggest that “benign” PTTs do not exist at all; rather, all PTTs are malignant, albeit with varying propensities for invasion [6]. Regardless of the semantic debate, it is critical to biopsy all PTTs and investigate for malignant behavior under histopathological examination.

The precise pathogenesis of MPTT remains to be elucidated, although Saida et al. proposed that MPTTs develop in a stepwise fashion, progressing from an adenomatous stage to an epitheliomatous stage before reaching a final malignant stage [4]. Sometimes, trauma or inflammation may incite malignant conversion of previously benign PTTs, although as in the case of our patient, they can also arise *de novo* [1].

The diagnosis of MPTT is challenging and MPTTs are most easily confused with squamous cell carcinoma. MPTTs can be distinguished from squamous cell carcinomas by the presence of a trichilemmal epithelium, which is not observed in squamous cell carcinomas. Additionally, the absence of an overlying precursor epidermal lesion (e.g. an actinic keratosis) favors a diagnosis of MPTT over squamous cell carcinoma in situ [7].

IHC has been proposed to be of utility in distinguishing MPTTs and squamous cell carcinomas [7]. However, based on our literature review, we suggest IHC be of limited value, as no IHC marker is consistently and specifically expressed to be reliably used in diagnosing MPTT (Table 1).

**Table 1:** Review of immunohistochemistry studies of malignant proliferating trichilemmal tumors Abbreviations: >, greater than; CD, cluster of differentiation; CK, cytokeratin; CR, current report; F, female; IHC, immunohistochemistry; M, male; NS, not stated; PHH3: phosphohistone H3; UEA-I: Ulex europaeus agglutinin-I ^a^Immunohistochemistry on the initial tumor showed 25% positivity for Ki-67. At three-year and four-year follow-up, the tumor showed 40% and 60% positivity for Ki-67, respectively.

Case	Patient Characteristics (Age, Sex)	Results of IHC	Reference
1	NS, NS	Positive for p53	Urano et al., 1992 [8]
2	57, M	Weakly positive for UEA-I	Ko et al., 1996 [9]
3	95, M	Weakly positive for UEA-I	Ko et al., 1996 [9]
4	56, F	Negative for UEA-I	Ko et al., 1996 [9]
5	32, M	Increasing Ki-67 positivity as the extent of the tumor advanced^a^	Park et al., 1997 [10]
6	64, F	Negative for CD34	Herrero et al., 1998 [11]
7	53, M	Negative for CD34	Herrero et al., 1998 [11]
8	66, F	Negative for CD34	Herrero et al., 1998 [11]
9	32, M	Positive for chromogranin A; positive for p53	Nakai et al., 2008 [12]
10	58, F	<1% CD34 reactivity; positive for Ki-67; strong diffuse nuclear positivity for p53	Chaichamnan et al., 2010 [13]
11	41, F	20% CD34 reactivity; positive for Ki-67; strong diffuse nuclear positivity for p53	Chaichamnan et al., 2010 [13]
12	65, F	CD34 positivity in >70% of tumor cells; Ki-67 positivity in 20% of tumor cells; strong diffuse nuclear positivity for p53; positive for calretinin	Gulati et al., 2011 [14]
13	52, F	40% CD34 reactivity; 20% Ki-67 reactivity; 80% p53 reactivity	Alici et al., 2015 [15]
14	67, M	Positive for Ki-67; positive for PHH3	Fieleke et al., 2015 [2]
15	42, M	Positive for CD34	Liu et al., 2016 [16]
16	46, F	Negative for CD34; negative for CK7; strongly positive for CK17; strong diffuse nuclear positivity for p53	CR

In particular, staining with CD34 has been touted as a diagnostic technique to distinguish MPTT from squamous cell carcinoma, with some investigators contending that MPTTs tend to be positive for CD34 while squamous cell carcinomas tend to be negative [7]. However, of the nine cases of MPTT stained with CD34, four (including our present case) were CD34 negative and one had less than 1% CD34 expression [11,13]. Thus, IHC studies with CD34 may actually be of limited value in clinching a diagnosis of MPTT. Nonetheless, as suggested by Chaichamanan et al., CD34 phenotype may correlate with the degree of tumor differentiation, with well-differentiated MPTTs being CD34 positive and poorly differentiated MPTTs being CD34 negative [13].

All seven of the MPTTs stained with p53 were positive for this marker. However, p53 is a highly non-specific marker that is positive in many other tumors, rendering it unreliable in differentiating MPTT and squamous cell carcinoma. IHC staining with chromogranin A, CK 7, CK 17, phosphohistone H3, Ulex europaeus agglutinin-I has also been reported, yet these markers are once again non-specific and thereby of limited diagnostic value.

Ki-67 is a similarly non-specific marker that is ubiquitously expressed in several cancers; however, like CD34, Ki-67 may be of salience in gauging the behavior of MPTTs. In the report by Park et al., the Ki-67 expression at initial evaluation was only 25%, yet at four-year follow-up (by which point the tumor had metastasized), the Ki-67 expression had risen to 60%, suggesting that Ki-67 positivity is correlated with tumor progression [10]. Of course, further studies are needed to establish this correlation. However, current literature suggests that strong Ki-67 positivity (in conjunction with weak CD34 positivity) may indicate a more aggressive MPTT requiring heightened vigilance.

There is no established paradigm of treatment for MPTTs. For MPTTs that have not metastasized, surgical excision with wide (1 centimeter) margins has been suggested [1]. Mohs surgery has been suggested as a superior alternative to simple resection as Mohs allows for more exquisite margin evaluation [2]. Radiation therapy has also been indicated for local tumors. For metastatic MPTTs, chemotherapy may be needed [1].

Even after complete extirpation, MPTTs can recur, emphasizing the need for follow-up [17]. Although no follow-up protocol for MPTTs has been established, an approach similar to the follow-up protocol for melanoma may be taken, with follow-up at three-month intervals for two years, then six-month intervals for three years. If no recurrence is noted at five years, then annual examinations may suffice. A computed tomography scan after initial diagnosis may also be warranted to evaluate for metastases.

## Conclusions

MPTTs classically present as rapidly growing masses accompanied by necrosis of underlying tissue. However, as in the case of our patient, they can present more subtly as ostensibly benign lesions, illustrating the need for biopsy and microscopic evaluation. Histopathological examination is crucial in establishing the diagnosis of MPTTs and reveals abrupt keratinization, anaplastic and pleomorphic cells, and increased mitotic forms. There are no IHC markers that are pathognomonic for MPTT, although CD34 and Ki-67 phenotypes may be of value in characterizing tumor behavior. Simple surgical resection with wide margins, Mohs surgery, and radiation therapy have all been reported in the management of local MPTTs that have not metastasized. A vigilant follow-up protocol may be warranted to monitor for recurrence.
